# Upregulation of circ_0000199 in circulating exosomes is associated with survival outcome in OSCC

**DOI:** 10.1038/s41598-020-70747-y

**Published:** 2020-08-13

**Authors:** Yanwei Luo, Fengxia Liu, Jie Guo, Rong Gui

**Affiliations:** 1 Department of Blood Transfusion, The Third Xiangya Hospital of Central South University, Tongzipo Road 138, Changsha, 410013 Hunan China; 2 National Institution of Drug Clinical Trial, Xiangya Hospital, Central South University, Xiangya Road 87, Changsha, 410008 Hunan P.R. China

**Keywords:** Cancer, Diseases, Molecular medicine, Oncology

## Abstract

Studies have found that circRNA in exosomes is associated with oral squamous cell carcinoma (OSCC) progression. In this study, we examined the expression of circ_0000199 in circulating exosomes from patients with OSCC and its role in the evaluation of relapse and prognosis. Real‐time quantitative reverse transcription–polymerase chain reaction was performed to assess circ_0000199 expression in circulating exosomes from 108 patients with OSCC and 50 healthy people. Gain- and loss-functional experiments were performed to assess the role of circ_0000199 on cell proliferation and apoptosis in OSCC cells. Our results showed that the high level of circ_0000199 in circulating exosomes was significantly associated with betel quid chewing, tumor size, lymphatic metastasis, and TNM stage in patients with OSCC. In addition, the patients with high exosomal circ_0000199 had higher tumor recurrence rate and higher mortality rate than the patients with low exosomal circ_0000199. Overexpression of circ_0000199 promoted, while knockdown of circ_0000199 inhibited OSCC cell growth. Bioinformatics analysis predicted that circ_0000199 interacted with miR-145-5p and miR-29b-3p simultaneously, which were involved in multiple tumor‐related signaling pathways. In conclusion, upregulation of circ_0000199 in circulating exosomes from patients with OSCC is positively associated with poor survival outcome. Circulating exosomal circ_0000199 can be used as a biomarker and potential therapeutic target for OSCC.

## Introduction

Oral squamous cell carcinoma (OSCC) is one of the most common malignant tumors in the world, accounting for 3% of malignant tumors^1^. Although the treatment of OSCC has made breakthroughs in the past decades, the 5-year mortality rate is still less than 50%^2^. Early diagnosis and early treatment are the keys to improve the survival rate of patients with OSCC.

Exosomes have phospholipid bilayer membrane with the markers TSG101 and CD63. The size of exosomes ranges from 30 to 100 nm. These vesicles exist in almost all body fluids of the human body, including blood, serum, saliva, cerebrospinal fluid, and urine^3^. Exosomes contain various substances, such as proteins, DNA, microRNA (miRNA), circular RNA (circRNA). These molecules can be transported to regulate the signaling pathways of the recipient cells^4^.

CircRNAs are covalently formed from the ends of a single RNA molecule, without 5′ and 3′ ribonucleotide end, and circRNAs are expressed abundantly, conserved and stably in cells with temporal and spatial specificity^5^. CircRNAs can bind to miRNAs to inhibit their functions. Therefore, circRNA is also called miRNA sponge. With the continuous understanding of circRNA, researchers have found that circRNAs are widely involved in the occurrence and development of physiology and disease as well as tumors^6^. As a new type of regulatory endogenous RNA, circRNAs are expected to become a tumor diagnostic marker and therapeutic target due to its characteristics such as stability, conservation, and tissue-specific expression^7^. For example, circRNA_100290 acts as a sponge for the miR-29 family, and activates CDK6 in oral cancer^8^. CircDOCK1 regulates BIRC3 expression and participates in the process of OSCC apoptosis^9^.

Studies have found that the level of circRNA in exosomes is closely related to oral cancer^10^. Has_circ_0000199 (circ_0000199) is a recently newly identified circular RNA, located at chr1: 243708811–243736350, derived from back splicing of exons 8–11 of AKT3. Circ_0000199 has been reported to be downregulated in clear cell renal cell carcinoma and glioblastoma, but upregulated in cisplatin-resistant gastric cancer tissues^11–13^, suggesting that circ_0000199 is tissue-specific. This study focused on the expression of circ_0000199 in circulating exosomes from patients with OSCC and its role in the evaluation of relapse and prognosis in patients with OSCC.

## Materials and methods

### Human sample collection

This study collected serum from 108 patients with OSCC and 50 healthy people from July 2014 to July 2019. All OSCC patients were confirmed by pathological diagnosis. Electronic health records, lifestyle, biochemical characteristics, tumor staging, treatment interventions, relapses, and survival time were recorded for all subjects. This study was approved by the ethics committee of the Third Xiangya Hospital, Central South University and in accordance with the 1964 Helsinki Declaration and its later amendments or comparable ethical standards. The written informed consents were obtained from all subjects.

### Exosomes isolation

The collected serum was centrifuged at 3,000×*g* for 15 min at 4 °C. The supernatant was filtered through a 0.22 μm filter, and exosomes were extracted using the ExoEasy Maxi Kit (cat no. 76064, Qiagen) according to the manufacturer’ instructions. The BCA kit (Thermo, USA) was used to quantify the exosome protein concentration, and the samples were aliquoted (100 μL each sample) and stored at – 80 °C.

### Exosomes identification

#### Transmission electron microscopy

5 μL of the exosome sample suspension was added to the Formvar-carbon copper mesh, and the exosome sample was stained with 3% phosphotungstic acid after being slightly dried. Exosomes were observed with a transmission electron microscope at 80 kV and electron micrographs were taken at 50,000×.

#### Exosome particle size analysis

The exosomes were resuspended in 50 μl of 1× PBS. The exosome particle size distribution was analyzed using ZetaView (Particle Metrix, Germany) according to the manufacturer's instructions.

#### The markers of exosomes

An Exosome Protein Extraction Kit (cat no. EZB-exo-PRO1, EZBioscience, Roseville, CA, USA) was used for protein extraction from exosomes according to the manufacturer recommended protocol. The proteins (20 μg) were separated on 12% SDSPAGE and transferred to a polyvinylidene fluoride (PVDF) membrane. The membranes were blocked with 5% skimmed milk at room temperature for 2 h. The membranes were then incubated with monoclonal primary antibody CD63 (1:1,000, Abcam) and TSG101 (1:1,000, Abcam), overnight at 4 °C. After washing 3 times with TBST, the membranes were incubated with secondary antibody goat anti-rabbit IgG antibody (1:2,000) for 2 h. The bands were developed with Immobilon Western HRP substrate (Millipore, USA). The original blots were presented in Supplementary Fig. 2.

### Cell culture

OSCC cell lines (SCC4, SCC9, SCC25, HN12, CAL27) and human oral keratinocyte cells (HOK) were purchased from the American Type Culture Collection (ATCC, Manassas, VA), and cultured in 37 °C incubator with 95% humidity and 5% CO_2_ concentration. The medium was changed every 2 days, and passaged every 4 days.

### Viral constructions and infection

The circ_0000199 vector was synthesized by Invitrogen Co., Ltd. circ_0000199 sequence was inserted into pcDNA3.1. Circ_0000199 without the downstream reverse sequence was used as a negative control. Circ_0000199 vector was finally cloned into the Tet-On Advanced Inducible Gene Expression System (Clontech Laboratories, Inc. Mountain View, CA, USA) according to the manufacturer's protocol. The target sequence of circ_0000199 small interfering RNA (siRNA) was 5′-TACTATTTTTCGACAAAAAGGTAAACAGC-3′. These adenoviruses were constructed using the AAVPrime AAV System (GeneCopoeia, Inc.) according to the manufacturer's protocol. SCC9 and HN12 were infected with viral at multiplicity of infection = 50 for 48 h.

### Quantitative polymerase chain reaction (qPCR)

QPCR was performed as previously described^14^. Total RNA was extracted from exosomes using Plasma/Serum Exosome Purification and RNA Isolation Mini Kit (cat no. 58300, Thorold, Canada) following the manufacturer recommended protocol. TaqMan Advanced miRNA assays (cat no. A25576, ThermoFisher, Shanghai, China) were used for detection of the expression of miRNAs following the manufacturer recommended protocol. SYBR Premix EX Taq II (TaKaRa) was used for detection of the expression of circRNA and mRNA by real-time quantitative PCR on ABI 7500 Real-Time PCR System (SeqGen, Inc., Torrance, CA). The primers were used as: circ_0000199, forward: 5′-CATTGCTTTCAGGGCTCTTGA-3′, reverse 5′-CCGCTCTCTCGACAAATGGA-3′; cytoplasmic polyadenylation element binding proteins (CPEB) 3, forward: 5′-TTTGCCAGAGCGGTCACATA-3′, reverse 5′-GTGCGGGAAGTTCTGGAAGA-3′; poly (A) nuclease 2 (PAN2), forward: 5′-CGCCCCAATTGTGGGTAACT-3′, reverse 5′- TTCAGGTGGGCATCCAAGAC-3′; , ring finger protein, LIM domain interacting (RLIM), forward: 5′-ACCCTAAAACCTAGTATTTTCCACT-3′, reverse 5′-AACGTCTTGCAGATGGCTCA-3′; , neurite extension and migration factor (NEXMIF), forward: 5′-TGTATCCAACATGGTGGCCC-3′, reverse 5′-TTGTGGACCTGTTCTCGCTC-3′; cofilin 2 (CFL2), forward: 5′-TGAGGCCGCCATTTTAACCT-3′, reverse 5′- CCAAGTGTCGAACGGTCCTT-3′; phosphatase and actin regulator 2 (PHACTR2), forward: 5′-GGACATGAACGCCTGGAAGT-3′, reverse 5′-CTTTCGGAGGCACAGGTGAT-3′; GAPDH, forward: 3′-GAAAGCCTGCCGGTGACTAA-5′, reverse 3′-TTCCCGTTCTCAGCCTTGAC-5′. GAPDH was used as an internal control.

### Cell viability assay

The cell viability was assessed by Cell Counting Kit-8 assay (cat no., B34304; Bimake, Shanghai, China) as previously described^15^. Briefly, SCC9 and HN12 cells were infected with circ_0000199 siRNA or circ_0000199 expressed adenoviruses for 48 h at 37 °C with 5% CO_2_, and then were plated in 96-well plates at a density of 1,000 cells in 100 μl of the aforementioned DMEM + FBS media per well. Ten μl of CCK8 reagent was added into each well and incubated for 1 h at 37 °C with 5% CO_2_. Absorbance at 450 nm was measured using the PARADIGM Detection Platform (Beckman Coulter, Inc., Brea, CA, USA).

### Terminal deoxynucleotidyl-transferase-mediated dUTP nick end labelling (TUNEL) assay

Apoptotic cells were analyzed using the TUNEL Chromogenic Apoptosis Detection kit (GeneCopoeia, Inc.) following the manufacturer recommended protocol.

### Bioinformatic analysis

Potential miRNA binding sites within circ_0000199 were predicted by miRNA target prediction software based on RNAhybrid and miRanda as previously described^16^. The potential targets of miR-145-5p and miR-29b-3p were predicted by miRwalk as previously described^17^. The structure of the miRNAs and target genes network were constructed using Cytoscape.

### Statistical analysis

All experiments were repeated at least three times, and data were expressed as the mean ± standard error of the mean. Graphpad prism software (version 8, GraphPad Software, Inc., San Diego, CA, USA) was used to perform statistical analysis. Differences between two groups were compared by student *t*-test. Differences among three or more groups were compared by one-way analysis of variance with a post hoc Bonferroni test. χ^2^ test was used to analyze categorical variables. Kaplan–Meier method was used to analyze the overall survival rate and relapse rate. P < 0.05 was considered to indicate a statistically significant difference.

## Results

### The expression of circ_0000199 in circulating exosomes of patients with OSCC

**Figure 1:**
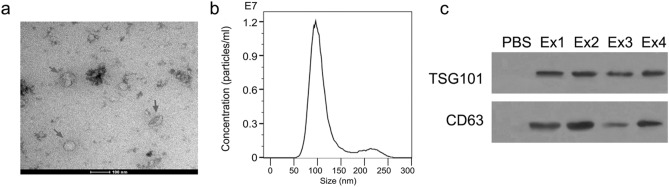
Identification of circulating exosomes. (**a**) The morphology of circulating exosomes (indicated by red arrow) was observed by transmission electron microscopy. (**b**) The exosome particle size distribution was analyzed using ZetaView. (**c**) Western blot was performed to test the markers of exosome, TSG101 and CD63. PBS was used as negative control. Ex, exosome. The original blots are presented in Supplementary Fig. 2.

**Figure 2:**
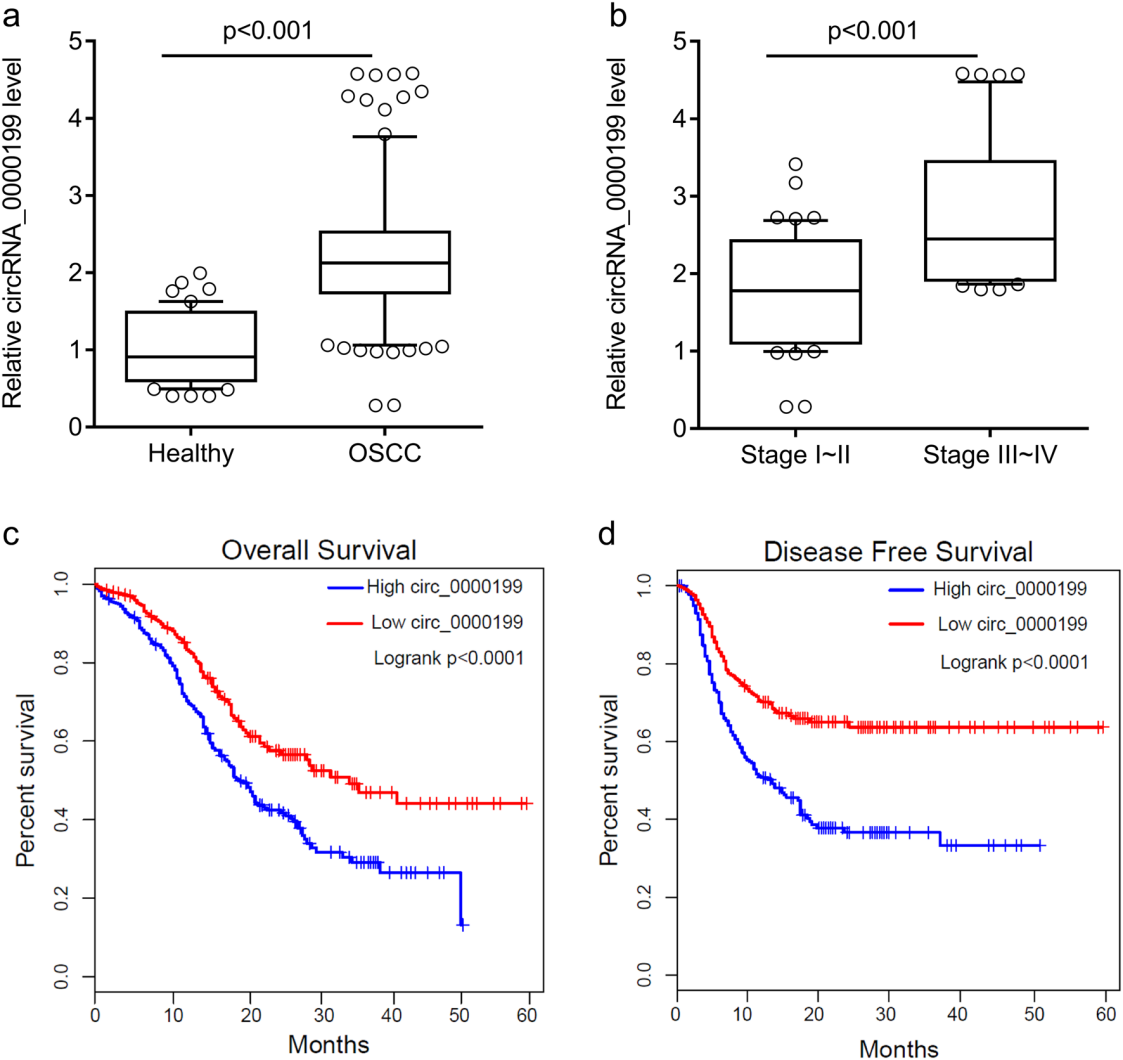
circ_0000199 is upregulated in circulating exosomes from patients with OSCC. (**a**) qRT-PCR analysis of expression levels of circ_0000199 in circulating exosome from patients with OSCC and healthy subjects. (**b**) the expression levels of circ_0000199 in circulating exosome in different TNM stage. OSCC, oral squamous cell carcinoma. (**c**) the overall survival rate was evaluated by Kaplan–Meier curve between OSCC patients with high or low circ_0000199 expression. (**d**) the disease-free survival rate was evaluated by Kaplan–Meier curve between OSCC patients with high or low circ_0000199 expression.

In this study, we isolated and purified circulating exosomes from 108 OSCC patients. The morphology of exosomes was identified by transmission electron microscopy (Fig. 1a). The size of particles was about 100 nm with concentration of 1.2 × 10^7^ particles/ml (Fig. 1b). These particles expressed the markers of exosomes TSG101 and CD63 (Fig. 1c), suggesting that we obtained the circulating exosomes from serum. Compared with the healthy subjects, the level of circ_0000199 in circulating exosomes from patients with OSCC was significantly increased (P < 0.001) (Fig. 2a), of which 68 cases with high expression of circ_0000199, and the other 40 cases with low expression of circ_0000199 compared with the mean in heathy subjects (Table 1). In OSCC patients, the level of circ_0000199 in the patients at III-IV TNM stage was significantly higher than in the patients with I–II TNM stage (P < 0.001) (Fig. 2b). We further analyzed the correlation between the level of circ_0000199 and the clinical characteristics of patients with OSCC. As shown in Table 1, the expression of circ_0000199 in circulating exosomes was significantly associated with betel quid chewing (P  = 0.0002), tumor size (P  = 0.0010), lymphatic metastasis (p = 0.0295), and TNM stage (P  = 0.0298). The expression of circ_0000199 in exosomes was not associated with gender, age, BMI, smoking, and tumor differentiation (Table 1).

**Table 1:** Association of tumor characteristics and circ_0000199 expression.

	High circ_0000199	Low circ_0000199	P value
N, %	68 (63.0)	40 (37.0)	
**Gender, N**			0.8039
Male	54	33	
Female	14	7	
Age, year, mean (SD)	59.5 (8.6)	61.3 (7.3)	0.2700
BMI, mean (SD)	22.3 (3.3)	21.7 (4.2)	0.4121
**Smoking history, N**			0.6117
Yes	57	32	
No	11	8	
**Betel quid chewing, N**			0.0002
Yes	59	21	
No	9	19	
Tumor size, cm, mean (SD)	6.4 (4.2)	3.8 (3.2)	0.0010
**Lymphatic metastasis, N**			0.0295
N0	29	26	
N1–3	39	14	
**Differentiation, N**			0.5135
Well	8	7	
Moderate	33	21	
Poor	27	12	
**TNM stage, N**			0.0298
I or II	33	28	
III or IV	35	12	

### Association between expression of exosomal circ_0000199 and survival outcome in patients with OSCC

In addition, we found that OSCC patients with high exosomal circ_0000199 had lower overall survival (P < 0.0001) and disease-free survival rate (P < 0.0001) than the patients with low exosomal circ_0000199 (Fig. 2c,d). Univariate and multivariate Cox regression analysis found that exosomal circ_0000199 was an independent factor affecting the survival of OSCC patients (HR, 3.57; 95% CI 2.48–6.24, P = 0.0035) (Tables 2, 3). In addition, the patients with high exosomal circ_0000199 had higher tumor recurrence rate (HR, 3.36; 95% CI 2.12–5.26, P = 0.0042) and higher mortality rate (HR, 4.31; 95% CI 2.57–7.28, P = 0.0027) than the patients with low exosomal circ_0000199 (Table 4).

**Table 2:** Univariate analysis of prognostic factors of OSCC.

Variable	Hazard ratio (95% CI)	P value
Gender (male vs. female)	1.03 (0.72–2.37)	0.2326
Age (≥ 60 vs. < 60)	1.12 (0.96–2.15)	0.5472
BMI (≥ 24 vs. < 24)	0.94 (0.63–1.78)	0.6436
Smoking history (yes vs. no)	1.37 (0.86–2.61)	0.5271
Areca nut use (yes vs. no)	2.53 (1.87–4.37)	0.0261
Tumor size (≥ 5 vs. < 5)	3.36 (2.68–4.75)	0.0152
Lymphatic metastasis (N1–3 vs. N0)	2.05 (1.79–3.63)	0.0374
Differentiation (moderate/poor vs. well)	1.12 (0.62–1.84)	0.7032
TNM stage (III-IV vs. I/II)	4.74 (2.84–6.56)	0.0031
Exosomal circ_0000199 (high vs. low)	3.07 (1.95–4.72)	0.0026

**Table 3:** Multivariate analysis of independent prognostic factors of OSCC.

Variable	Hazard ratio (95% CI)	P value
Areca nut use (yes vs. no)	2.15 (1.64–4.05)	0.0135
Tumor size (≥ 5 vs. < 5)	2.61 (1.58–4.87)	0.0204
Lymphatic metastasis (N1–3 vs. N0)	1.84 (1.36–3.28)	0.0239
TNM stage (III/IV vs. I/II)	4.47 (2.37–7.12)	0.0007
Exosomal circ_0000199 (high vs. low)	3.57 (2.48–6.24)	0.0035

**Table 4:** Association of recurrence and mortality and circ_0000199 expression.

	1 year	3 year	5 year	HR (95% CI)	P value
**Recurrence, %**					
High circ_0000199	21.6	35.4	68.3	3.36 (2.12–5.26)	0.0042
Low circ_0000199	11.2	22.3	37.5		
**Mortality, %**					
High circ_0000199	18.2	33.6	74.2	4.31 (2.57–7.28)	0.0027
Low circ_0000199	7.5	21.8	42.6		

### Biological function of circ_0000199 in OSCC cells

**Figure 3:**
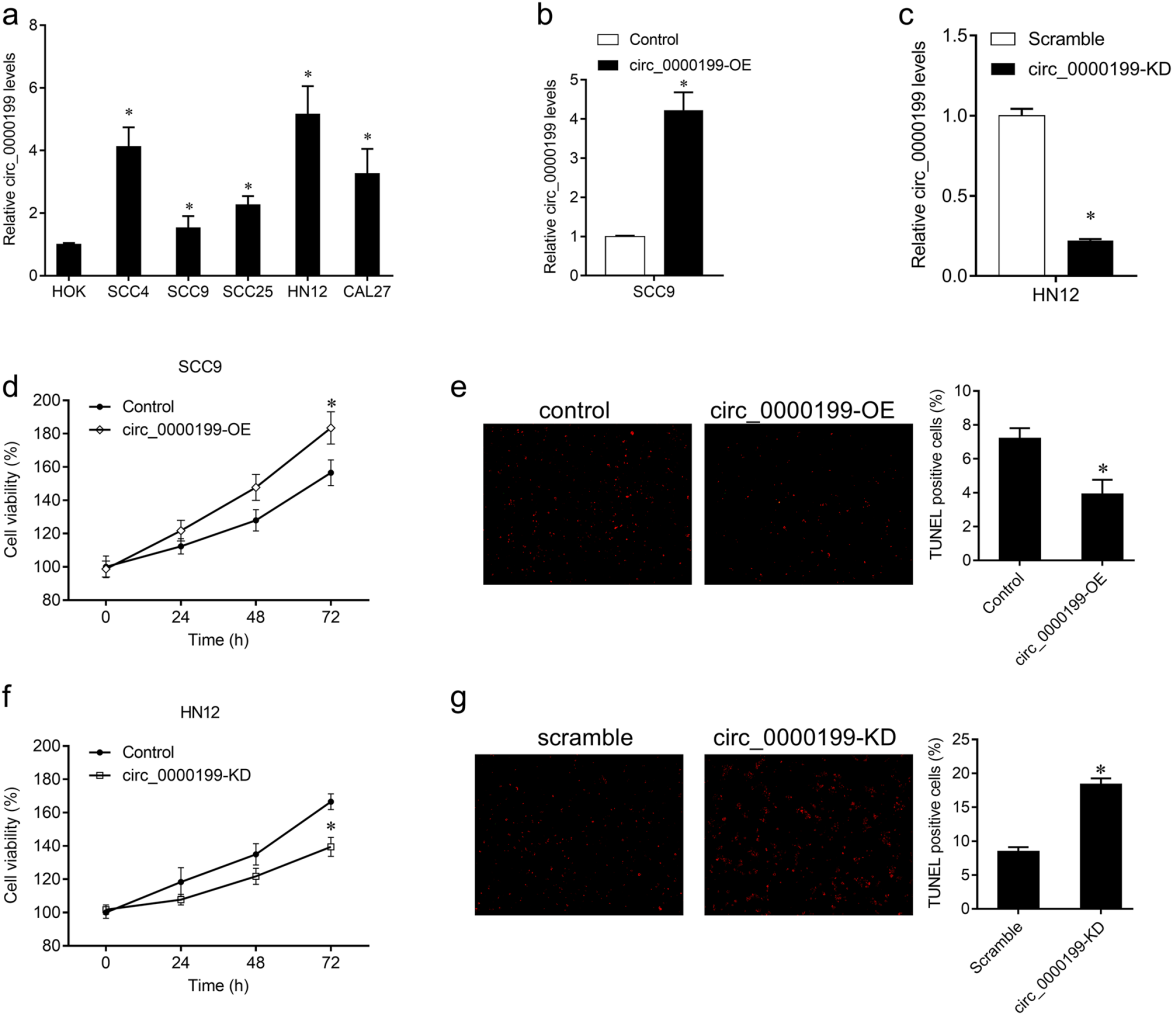
The effects of circ_0000199 on OSCC cells. (**a**) qRT-PCR analysis of expression levels of circ_0000199 in OSCC cell lines and HOK cells. (**b**) qRT-PCR analysis of expression levels of circ_0000199 in SCC9 cells after infection with virus expressed circ_0000199. (**c**) qRT-PCR analysis of expression levels of circ_0000199 in HN12 cells after infection with virus containing circ_0000199 siRNA. (**d**) CCK8 was performed to measure cell viability after infection with virus expressed circ_0000199 in SCC9 cells. (**e**) TUNEL staining was performed to measure apoptotic cells after infection with virus expressed circ_0000199 in SCC9 cells. (**f**) CCK8 was performed to measure cell viability after infection with virus containing circ_0000199 siRNA in HN12 cells. (**g**) TUNEL staining was performed to measure apoptotic cells after infection with virus containing circ_0000199 siRNA in HN12 cells. *P < 0.05.

To further study the role of circ_0000199 in OSCC cells, we tested the expression of circ_0000199 in different OSCC cell lines. Compared with HOK cells, the expression of circ_0000199 in OSCC cells was significantly increased, with the highest expression in HN12 and the relative lower expression in SCC9 cells (Fig. 3a). We selected SCC9 and HN12 cells for further analysis. Circ_0000199 was overexpressed in SCC9 cells, and knocked down in HN12 cells (Fig. 3b,c). The effects of circ_0000199 on the proliferation and apoptosis of OSCC cells were studied by CCK8 and Tunel staining. The results showed that overexpression of circ_0000199 in SCC9 cells significantly promoted cell proliferation and reduced the number of TUNEL-positive cells (Fig. 3d,e); while knockdown of circ_0000199 in HN12 cells significantly inhibited cell proliferation and increased the number of TUNEL-positive cells (Fig. 3f,g).

**Figure 4:**
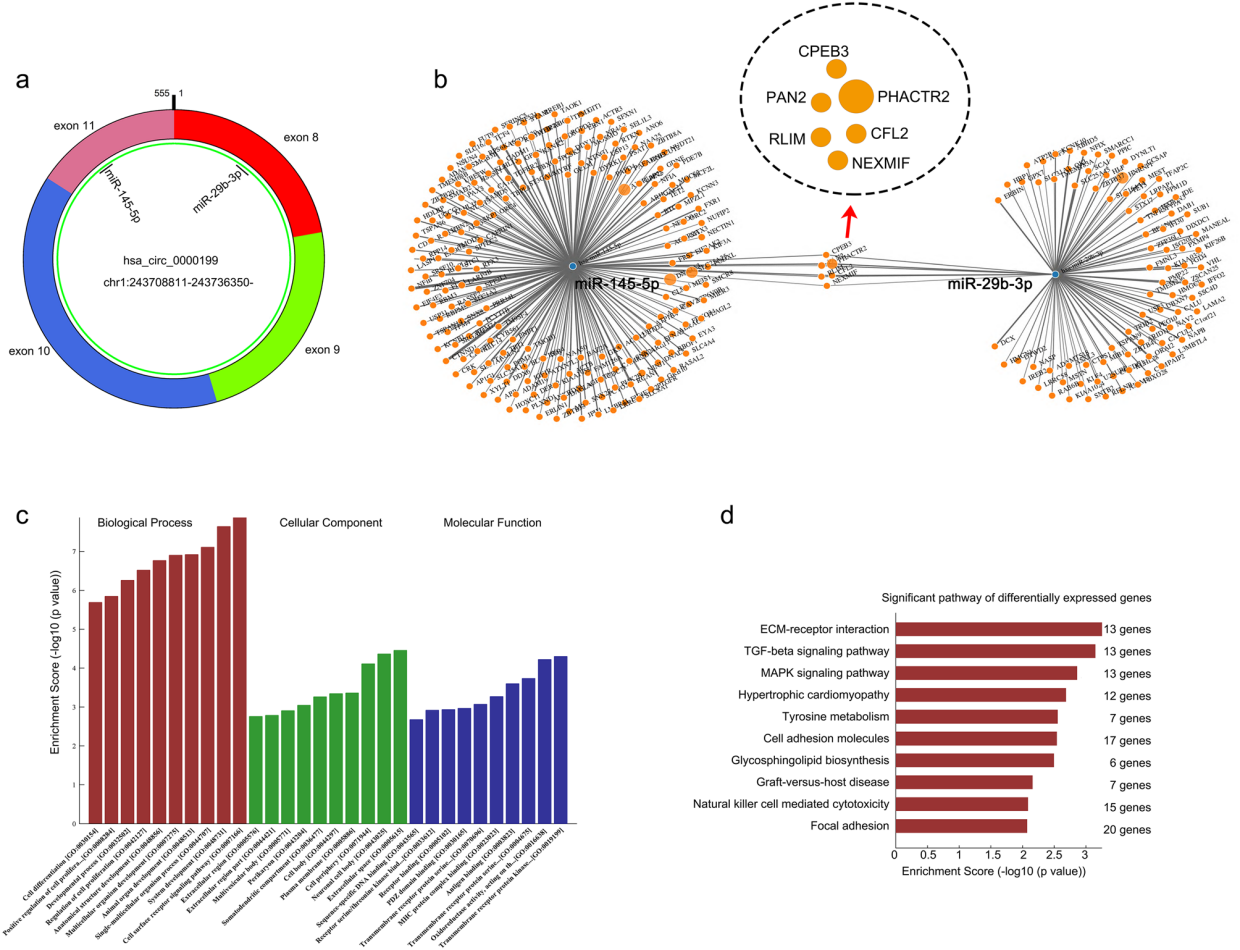
The downstream molecules of circ_0000199. (**a**) Diagram showing the binding sites for miR‐145‐5p and miR‐29b‐3p in hsa_circ_0072387. (**b**) The mRNA–miRNA gene co‐expression network. (**c**) The bar chart shows the top 10 enrichment signaling in biology process, cellular component, molecular function. (**d**) The bar chart shows the top 10 enrichment KEGG pathways.

We further studied the mechanism of circ_0000199. Bioinformatics analysis revealed that circ_0000199 could interact with miR-145-5p and miR-29b-3p simultaneously (Fig. 4a). We also found that compared with HOK cells, the expression of miR-145-5p in OSCC cells was significantly decreased, with the lowest expression in HN12 and the relative higher expression in SCC9 cells; similar results were also observed in the expression of miR-29b-3p (supplementary Fig. 1a). In addition, the prediction of downstream target genes for miR-145-5p and miR-29b-3p revealed 5 common target genes (including CPEB3, PAN2, RLIM, NEXMIF, CFL2, and PHACTR2) (Fig. 4b). We then examined the expression of these target genes after overexpression or knockdown of circ_0000199 in SCC9 and HN12 cells, respectively. We found that overexpression of circ_0000199 significantly increased the expression of CPEB3, PAN2, RLIM and CFL2 in SCC9 cells, while knockdown of circ_0000199 significantly inhibited the expression of CPEB3, PAN2, RLIM and CFL2 in HN12 cells (supplementary Fig. 1b). However, overexpression and knockdown of circ_0000199 did not significantly alter the expression of NEXMIF and PHACTR2 in SCC9 and HN12 cells (supplementary Fig. 1b). These target genes together affected a variety of biology processes, cellular component and molecular functions (Fig. 4c). KEGG database was used to analyze the pathways that were enriched by these target genes^18^. The results showed that ECM-receptor interaction and TGF-beta signaling pathway may be the pathways with the greatest enrichment (Fig. 4d).

## Discussion

Oral squamous cell carcinoma is a common head and neck tumor. Early surgery or radiotherapy can achieve good results, but most patients with head and neck squamous cell carcinoma have exhibited cervical lymph node metastasis or distant metastasis at the first diagnosis. Although surgery plus radiotherapy or chemotherapy can delay the progression of the disease, the patients with stage III/IV do not be improved the long-term survival rate^19^. Therefore, early diagnosis and early treatment are the key to the treatment of OSCC. Recent studies have found that circulating exosomes in oral cancer patients are significantly higher than healthy people, which may be involved in the occurrence and development of oral cancer^20^. Exosomes contain proteins, nucleic acids, and lipids. By passing these connotative substances between cells, exosomes can regulate immune function and promote tumor angiogenesis and metastasis, even directly affect tumor cell growth^21,22^.

CircRNAs are often enriched in exosomes and have high stability and tissue specificity. Therefore, exosomal circRNAs may be useful markers for diagnosis and treatment of OSCC. In this study, circulating exosomal circ_0000199 was significantly increased in patients with OSCC and increased with the TNM stage. In addition, high expression of circ_0000199 was associated with betel quid chewing, tumor size, and lymph node metastasis, suggesting its potential clinical application as an independent factor for predicting survival and disease recurrence of OSCC patients. The epidemiological studies have showed that betel quid chewing is closely related to the occurrence of OSCC^23^. In 2003, the World Health Organization identified betel nut as a primary carcinogen. Areca nut contains various alkaloids, including arecoline (ARC), arecaidine, and guvacoline. Among them, ARC has the closest relationship with OSCC. ARC has cytotoxic effects on oral mucosal fibroblasts, can promote cell proliferation, induce collagen synthesis, cause collagen accumulation and induce oral submucous fibrosis, and further accumulation may lead to the occurrence of OSCC^24^.

By gain- and loss-functional experiments, we further found that overexpression of circ_0000199 was able to promote cell proliferation and inhibit apoptosis, while knockdown of circ_0000199 exhibited opposite effects. Previous studies have shown that circRNAs, as a new type of competitive endogenous RNA (ceRNA), act as miRNA sponge by which interact with miRNA to cause dysregulated expression of miRNA target genes, thereby participating in OSCC process^25,26^. In this study, bioinformatics software predicted that circ_0000199 could interact with miR-145-5p and miR-29b-3p, which were downregulated in OSCC cells. MiR-145 and miR-29 are both tumor suppressor genes in OSCC. Shao et al. found that miR-145 inhibited the growth of OSCC cells by targeting c-Myc and CDK6^27^, while miR-29-3p was also a predicted target of circ_0072387 in oral squamous cell carcinoma cells^28^. Further analysis revealed that miR-145-5p and miR-29b-3p could jointly target CPEB3, PAN2, RLIM, NEXMIF, CFL2 and PHACTR2. In addition, circ_0000199 could positively regulated the expression of CPEB3, PAN2, RLIM and CFL2 in OSCC cells. Studies have showed that aberrant expression of these proteins correlates with certain types of cancer. CPEBs are associated with specific sequences in mRNA 3 'untranslated regions to promote translation. Several CPEBs govern cell cycle progression, regulate senescence, establish cell polarity, and promote tumorigenesis and metastasis^29^. PAN2 is an important regulator of the HIF1A-mediated hypoxic response^30^. RLIM knockdown significantly inhibits the proliferative and migratory capacities of prostate cancer cells^31^. High CFL2 expression is associated with poor overall survival in gastric cancer^32^. Therefore, miR-145-5p and miR-29b-3p play an important role in tumorigenesis and development of OSCC. Through analysis of the functions and signaling pathways of downstream molecules, we found that circ_0000199 had the greatest effect on cell surface receptor signaling pathway in biology process, extra cellular space in cellular components, and transmembrane receptor protein kinase in molecular functions. In addition, the major downstream signaling pathways regulated by circ_0000199 were ECM-receptor interaction, TGF-beta signaling pathway and MAPK signaling pathway. These signaling pathways have important effects on the proliferation and apoptosis of OSCC cells^33,34^, suggesting that circ_0000199 regulates these signaling pathways via direct or indirect manner, and ultimately affects the progress of OSCC, but the specific regulatory molecular mechanism still needs more experiments to illustrate.

In summary, circulating exosomal circ_0000199 is highly expressed in patients with OSCC and acts as an independent predictor for survival and disease recurrence in patients with OSCC. In addition, circ_0000199 is an oncogene in OSCC cells. These findings suggest that circulating exosomal circ_0000199 can be used as biomarker and potential therapeutic target for OSCC. However, more studies, with more patients are need to confirm these findings.

## Supplementary information

Supplementary Information 1.

## Data Availability

All data generated or analysed during this study are included in this published article.
