# Supplementary material for: Upregulation of circ_0000199 in circulating exosomes is associated with survival outcome in OSCC
Source: Sci Rep. 2020 Aug 13;10:13739. doi: 10.1038/s41598-020-70747-y (PMC7426867; doi:10.1038/s41598-020-70747-y)
Supplement: Supplementary file 1 — Supplementary Information 1. [file 41598_2020_70747_MOESM1_ESM.pdf]

Upregulation of circ_0000199 in circulating exosomes is associated with survival outcome in OSCC

Yanwei Luo[1], Fengxia Liu[1], Jie Guo[2]*, Rong Gui[1]*

1.Department of Blood Transfusion, the Third Xiangya Hospital of Central South University, Changsha, China, 410013

2.National Institution of Drug Clinical Trial, Xiangya Hospital, Central South University, Changsha 410008, Hunan, P.R. China.

*Corresponding author: Dr. Jie Guo

Department: National Institution of Drug Clinical Trial, Xiangya Hospital, Central South University

Address: Xiangya Road 87, Changsha 410008, Hunan, P.R. China,

Tel./fax: 86-073184327458

E-mail: xiangyaguojie@hotmail.com

Prof. Rong Gui,

Department of Blood Transfusion, the Third Xiangya Hospital of Central South University, Changsha, Hunan, China.

Address: Tongzipo Road 138, Changsha, Hunan, 410013, China;

Phone/Fax: +86-073188618513

E-mail: aguirong@163.com

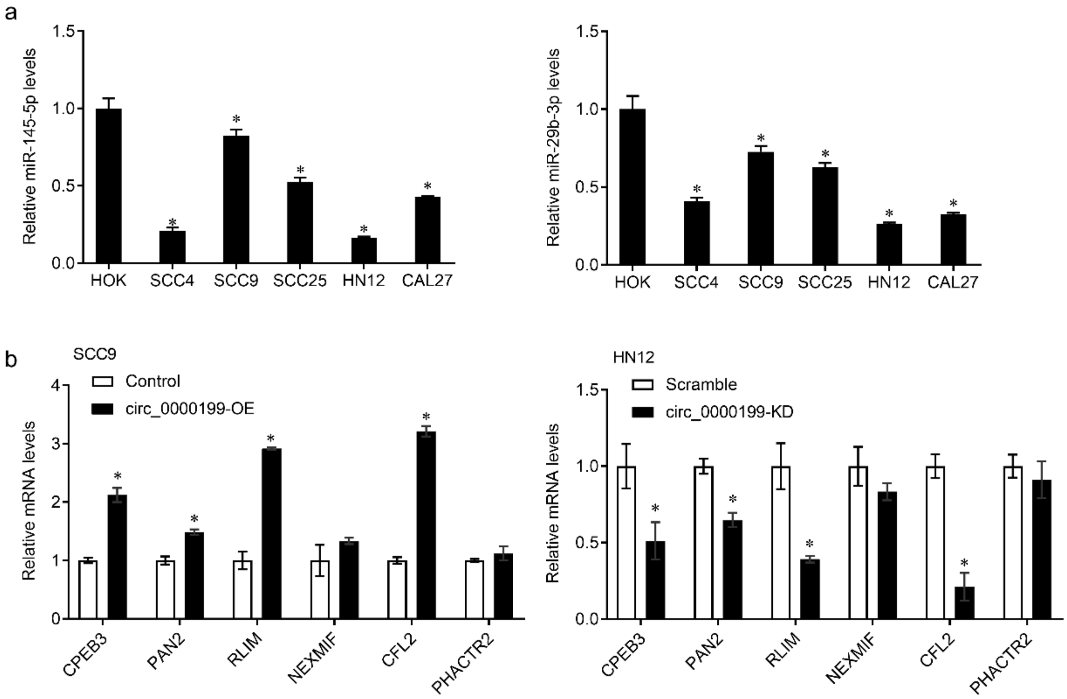

Supplementary Figure 1 The expression of miRNAs and their target genes. (a) qRT-PCR analysis of expression levels of miR-145-5p and miR-29b-3p in OSCC cell lines and HOK cells. (b) qRT-PCR analysis of mRNA levels of target genes in SCC9 cells after infection with virus expressed circ_0000199 (left), or in HN12 cells after infection with virus containing circ_0000199 siRNA (right).

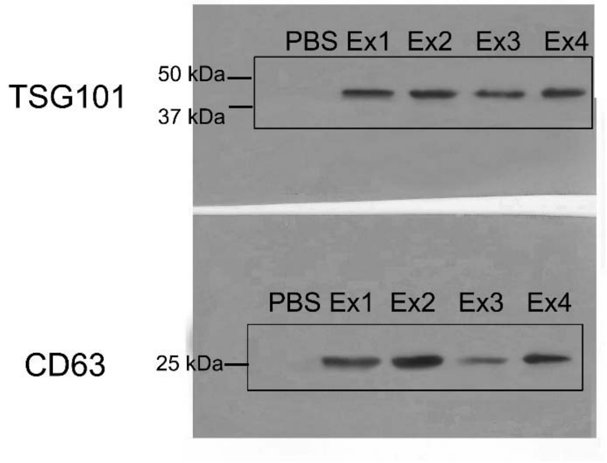

Supplementary Figure 2 The original blots of TSG101 and CD63 in Figure 1c. Western blot was performed to test the markers of exosome, TSG101 and CD63. PBS was used as negative control. Ex, exosome.
